# Editorial: Assessment of osteoporotic fractures and risk prediction

**DOI:** 10.3389/fendo.2022.1107678

**Published:** 2022-12-19

**Authors:** Xiaoguang Cheng, Zhi-Feng Sheng, Xiangbing Wang

**Affiliations:** ^1^ Department of radiology, Beijing Jishuitan Hospital, Beijing, China; ^2^ Second Xiangya Hospital, Central South University Changsha, Changsha, China; ^3^ The State University of New Jersey, New Brunswick, NJ, United States

**Keywords:** osteoporosis, fracture, risk factors, prediction, assessment

Osteoporosis is a metabolic skeletal disorder that is characterized by low bone mineral density (BMD), a deterioration of the microstructure of bone tissue, and a decrease in bone strength, leading to an increase in bone fragility and the risk of fractures (1). Symptomatic vertebral and hip fragility fractures are severe osteoporotic fractures that limit the quality of life and increase morbidity and mortality [(2), Shen et al, ( 3)]. Currently, a total of 10.9 million men and 49.3 million women in China are estimated to have osteoporosis (4). Meanwhile, it has been estimated that world–wide, there were 158 million individuals aged 50 years or older at high fracture risk in 2010, and that number is expected to double by 2040, predominantly in Asia (5). Therefore, early screening for osteoporosis has a significant role in controlling the disease and lowering the prevalence of osteoporotic fractures.

Although great advances have been achieved in surgical strategies for the treatment of osteoporotic fractures, information on the early assessment of osteoporotic fractures remains limited. Therefore, we organized this special issue that aims to provide insight into the etiology and pathogenesis of osteoporotic fractures, such as the connections between bone mineral density, bone mineral content, and muscle, focusing on clinical research related to the diagnosis, prevention, treatment, and monitoring of osteoporotic fracture. We received more contributions on this topic than originally anticipated, so we have expanded the special issue into a two–volume collection.

Among the contributions in this collection, a retrospective study by Li and colleagues provides clear evidence that modifiable body composition indicators such as body mass index (BMI), body fat percentage (BFP), and skeletal muscle index (SMI) are significantly associated with osteoporosis (6). In a study of the relationships between anthropometric variables and osteoporotic fracture risk, Wu et al. report that body surface area (BSA) may be a potential new risk factor for osteoporotic fractures (7). Moreover, based on their BSA stratification, the authors conclude that BSA may be a risk factor for clinically severe osteoporotic fractures in men with the risk significantly increased by 41–55% when BSA ≤ 1.6895 m^2^. Regarding vertebral fractures, Liu et al. have investigated the prevalence of vertebral fractures in middle–aged and elderly Chinese individuals (8). Based on the China Action on Spine and Hip Status (CASH) study, the authors concluded that the prevalence of vertebral fractures increased rapidly in women after age 50, but comparatively slowly in men. In addition, participants under the age of 50 with a grade 1 vertebral fracture had normal bone mass compared with non–fractured participants (6). The authors’ conclusions are consistent with another recently published report (9). In a study of hip fractures, Wang et al. found substantial differences in total and cortical volume as well as cortical thickness between fractured and non–fractured women across the proximal femur. The study of three–dimensional bone geometry and soft tissue is of particular interest in hip fracture research (10–12). Mao et al. have constructed a convolutional neural network model for screening primary osteopenia and osteoporosis based on lumbar radiographs, which may help improve the low rate of diagnosis of osteoporosis (13). Kou et al. have investigated possible diagnostic markers for the early diagnosis of osteoporosis on untargeted gas chromatography (GC)/liquid chromatography (LC)–mass spectrometry (MS) and identified 18 differential metabolites that are potential biomarkers of osteoporosis in postmenopausal women.

Other studies in this special issue investigated risk factors affecting bone mineral density, such as hyperglycemia [Wang et al.], serum amino acid levels [Cui et al.], non–alcoholic fatty liver disease and the degree of hepatic steatosis [Xie and Liu], MicroRNAs in Serum Exosomes [Shi et al.], milk intake [Chen et al.], Neuropeptide Y [Chen and Zhang], nitrates [Liu et al.], menopause–related cortical bone loss (14).

In conclusion, the articles included in this two–volume collection offer fresh perspectives into the etiology and pathogenesis of osteoporotic fractures. With more research in this critical area, we anticipate that many of these discoveries will find their way into clinical practice.

## Author contributions

XC, Z–FS, and XW contributed to conception of the study. XC wrote the first draft of the manuscript. All authors contributed to manuscript revision, read, and approved the submitted version.
